# MR-Guided Transurethral Ultrasound Ablation (TULSA)—An Emerging Minimally Invasive Treatment Option for Localised Prostate Cancer

**DOI:** 10.1007/s00270-024-03696-y

**Published:** 2024-03-15

**Authors:** Kin Fen Kevin Fung, Roberto Luigi Cazzato, Thibault Tricard, Pierre D. E. Marini, Gregory Bertucci, Pierre-Alexis Autrusseau, Guillaume Koch, Julia Weiss, Julien Garnon, Hervé Lang, Afshin Gangi

**Affiliations:** 1 Department of Radiology, University of Hong Kong, Hong Kong, Hong Kong; 2 Department of Interventional Radiology, University Hospital Strasbourg, Strasbourg, France; 3 Department of Urology, University Hospital Strasbourg, Strasbourg, France; 4 Department of Human Anatomy, University of Strasbourg, Strasbourg, France; 5 Department of Radiology, Hong Kong Children’s Hospital, Hong Kong, Hong Kong; 6 School of Biomedical Engineering and Imaging Sciences, King’s College London, London, UK

**Keywords:** Prostate cancer, Ablation, Interventional oncology, Magnetic resonance

## Abstract

The optimal treatment strategy for men with localised prostatic cancer of low and intermediate risk is an actively evolving field. It is important to strike a balance between maximal oncological control and minimal treatment-related complications, which helps preserve the patients’ quality of life. MR-guided transurethral ultrasound ablation (TULSA) has emerged as a minimally invasive treatment option for this group of patients. This article aims to provide of a background on TULSA technology, a step-by-step procedural guide of MR-guided TULSA and to summarise the current evidence of TULSA in management of localised prostatic cancer, as well as other potential indications.

**Graphical Abstract:**

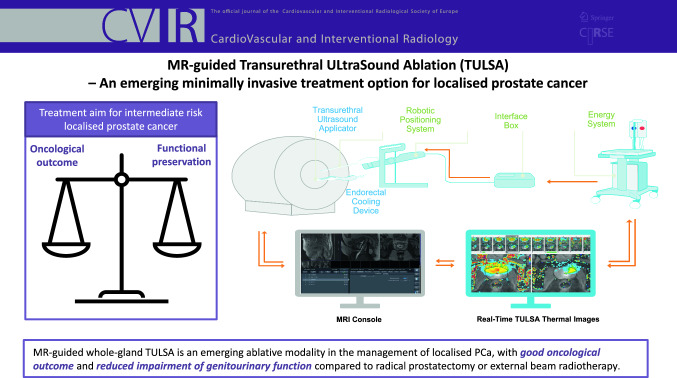

**Supplementary Information:**

The online version contains supplementary material available at 10.1007/s00270-024-03696-y.

## Introduction

Worldwide, prostate cancer (PCa) is the second most common cancer in men [1]. With increasing public awareness of the disease, use of prostate specific antigen (PSA) screening and availability of advanced imaging modality such as multiparametric prostate MRI, there has been a trend towards earlier diagnosis of clinically significant localised PCa in lower risk groups [2]. Conventional treatments including radical prostatectomy, external beam radiotherapy and brachytherapy are currently considered the standard curative management in patients with intermediate-risk localised PCa and life expectancy greater than 10 years. However, these treatments carry significant side effects such as erectile and urinary incontinence, which can negatively impact the patient’s quality of life. Therefore, for low- and intermediate-risk patients, the strategy of active surveillance (AS) is recommended in current guidelines to avoid over-treatment [3]. AS typically involves a structured monitoring program using PSA, repeated biopsies and MRI to identify patients with disease progression. However, 10% of patients experience significant anxiety whilst on AS and would opt for curative treatment [4]. The American Urological Association/American Society for Radiation Oncology guidelines for localised PCa recommends that whole-gland or focal ablation may be considered in patients with intermediate-risk PCa [5].

High-intensity focused ultrasound (HIFU) and cryoablation are the most commonly investigated ablative modalities for low to intermediate-risk localised PCa [6–8]. These modalities utilise thermal energy, often delivered under image guidance, to destroy prostate tissue harbouring cancer cells [9]. The aim of ablation is to achieve equivalent oncological outcome with reduced genitourinary complications, when comparing to conventional surgery or radiotherapy. Whole-gland ablation was conventionally investigated as the alternative to radical prostatectomy or external beam radiotherapy, which aims to treat the entire gland, regardless of the location of tumour within the prostate. In patients with unifocal and/or unilateral localised PCa, focal ablation was studied as a possible treatment option with reduced treatment-related functional disturbances [10]. In patients with multifocal localised PCa, with the aid of multiparametric MRI and fusion technology, focal ablation can also target the “index tumour”, i.e. the lesion that is of the highest grade and biologically most aggressive, while sparing the surrounding prostate tissue, urinary sphincter and rectum [11]. However, both HIFU and cryotherapy have their limitations. In terms of whole-gland ablation, HIFU can only ablate prostate gland with volume up to 40 mL and cyroablation up to 60 mL. For focal ablation, HIFU is less effective in treating anteriorly located tumour due to the dissipation of energy from posteriorly located rectal probe [12].

In recent years, MR-guided transurethral ultrasound ablation (TULSA) has emerged as a novel focal or whole-gland treatment option for patients with localised PCa, especially in low- and intermediate-risk groups [13–17]. This article aims to review the scientific basis of MR-guided TULSA, procedural details, current evidence in the literature supporting its use in localised prostate cancer, as well as the way forward.

## TULSA technology and mechanism of action

**Fig. 1:**
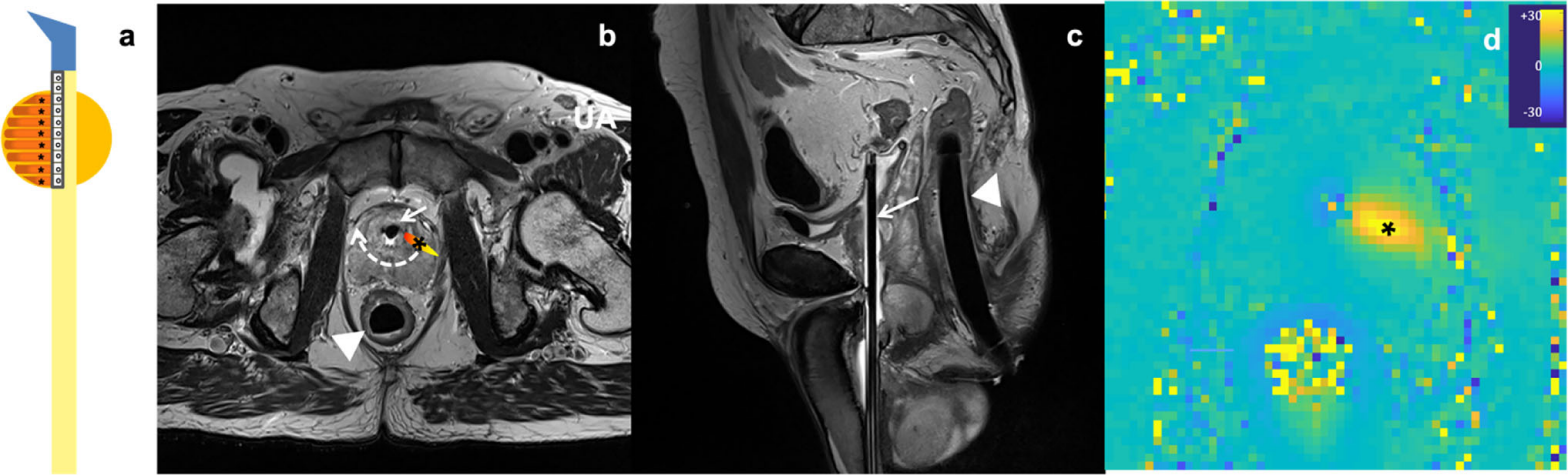
MRI-guided TULSA is a minimally invasive ablation technique providing thermal-mediated destruction of the prostate. **a** The rigid rotational transurethral ultrasound applicator delivers a directed, non-focused ultrasound energy (*) to the prostate (yellow circle) via ten independent ultrasound transducers (°). **b**, **c** Axial and sagittal T2W images show in situ position of transurethral ultrasound applicator (white arrow) and endorectal cooling device (white arrowhead). Dashed white arrow indicates the rotational sweep of directional ultrasound energy (*). **d** Real-time MR thermometry is continuously acquired to provide a prostatic temperature map, which is fed back to a dedicated algorithm to automatically adjusts the frequency and power of each UA transducer

TULSA delivers a directed non-focused ultrasound energy (4–14 MHz) to the prostate through a rigid rotational transurethral ultrasound applicator (UA) consisting of ten independent ultrasound transducers (Fig. 1). Prostatic tissue is heated to 57 °C, resulting in coagulative necrosis. Compared to transrectal high-intensity focused ultrasound (HIFU), the directional ultrasound (US) beam of TULSA can interact with a larger volume of prostatic tissue, resulting in shorter treatment time and larger ablation zone (100 mL in TULSA vs. 40 mL in HIFU). In addition, the angular sweep of TULSA US beam can also be adjusted in order to deliver focal/partial or whole-gland ablation [18].

Real-time MR thermometry is continuously acquired during the ablation to provide a temperature map of the prostate. Based on this thermal map, a closed-loop temperature feedback control algorithm will then control the frequency and power of each UA transducer, as well as the rotational speed of the UA. This enables precise ablation of target prostatic tissue under real-time monitoring and can avoid excessive heat deposition in periprostatic structures (rectum, urinary sphincter, bladder neck and neurovascular bundles) to prevent complications. Continuous cooling of the urethra (through the UA) and the rectum (through an endorectal cooling device) also helps prevent thermal injuries to these structures.

## MR-guided TULSA procedure

**Fig. 2:**
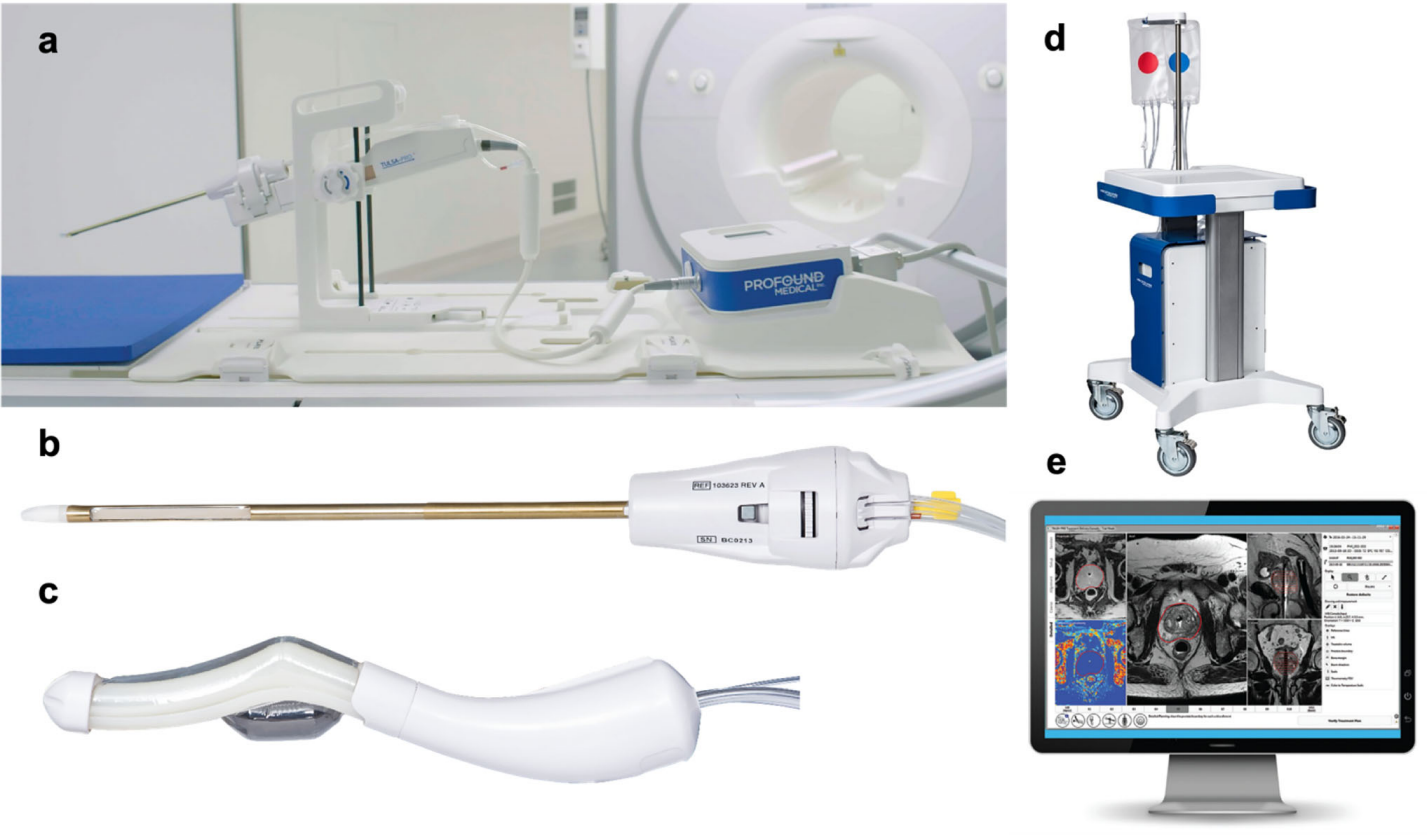
Components of the MR-guided TULSA-PRO system. **a** Robotic positioning system, **b** Urethral applicator, **c** Endorectal cooling device, **d** Energy system with coolant fluids, **e** Treatment delivery console

**Fig. 3:**
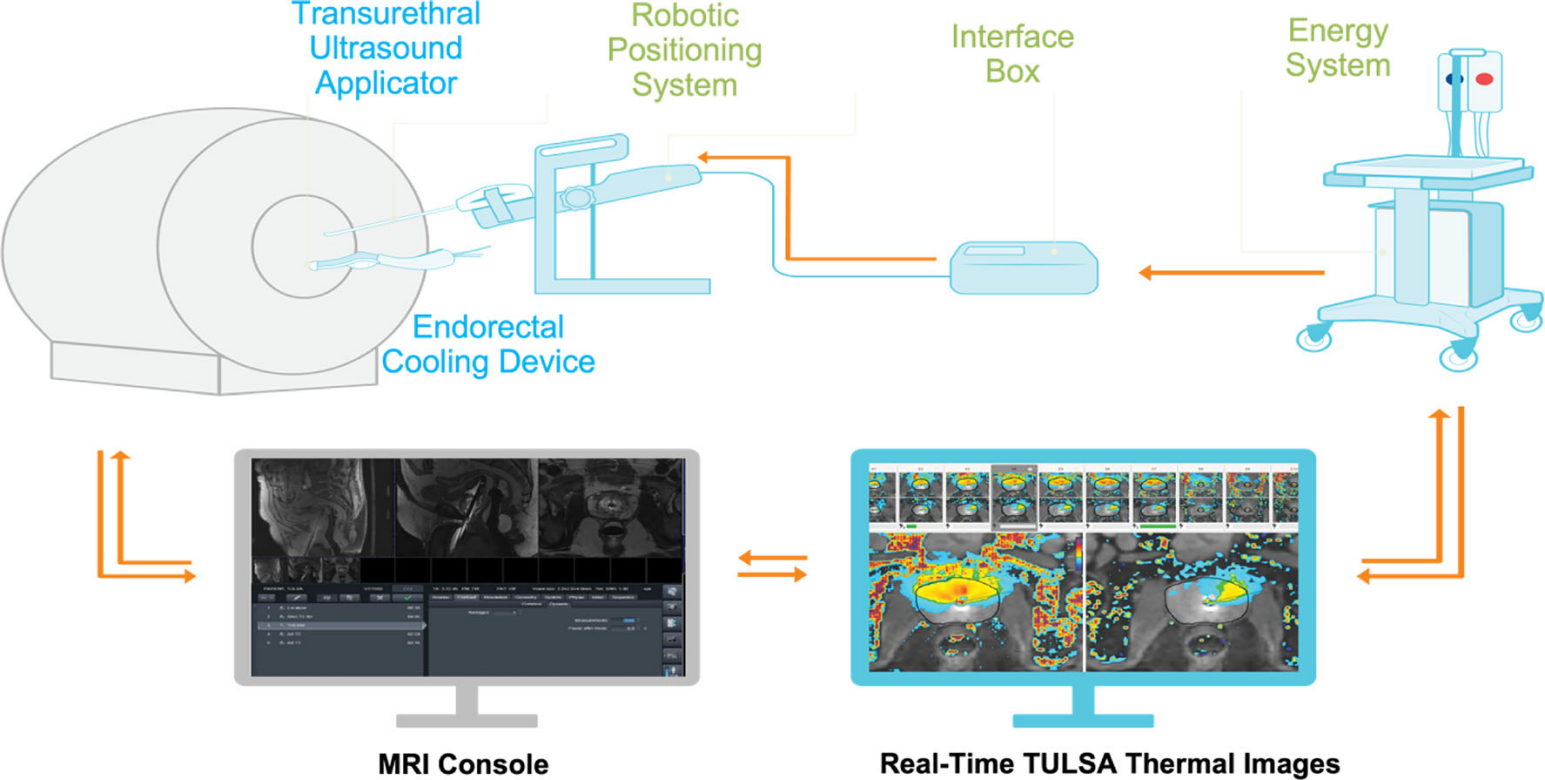
Diagrammatic illustration shows how the TULSA-PRO system functions. The robotic positioning system holds the urethral applicator in place, allowing remote linear and rotational motion within the prostatic urethra. The energy generator and cooling system ensures continuous infusion of coolant solution to urethral applicator and endorectal cooling device, which protect the periurethral tissue and rectal wall. The radiologist can perform device registration, treatment planning and delivery on the treatment delivery console, where real-time MR thermometry is displayed

TULSA-PRO system (Profound Medical, Mississauga, Canada) is FDA-approved, CE-marked for MR-guided prostate gland ablation. The components and diagrammatic illustration of system are outlined in Figs. 2 and 3, respectively.

The procedure consists of four stages: preparation, planning, delivery and confirmation.

- (a) Preparation

The patient is positioned supine in a low lithotomy position under general anaesthesia in the MR suite. The urinary bladder is first catheterised using a Foley’s catheter and a straight tip 0.038-inch stiff non-hydrophilic guidewire (Boston Scientific, Marlborough, Massachusetts). The Foley’s catheter would then be exchanged for the rigid rotational UA. The endorectal cooling device (ECD) would also be placed inside the patient’s rectum. A robotic positioning system holds the UA in place to provide remote linear and rotational motion of the device within the prostatic urethra.

Survey MR images using localiser or T2-weighed sequence are acquired to confirm the position of UA and ECD. The active zone of the UA is indicated by two white dots. It is important that there is no air or faecal material interposed between the rectal wall and ECD to ensure adequate cooling of rectal wall. The concavity of ECD should also align with the prostatic curvature for satisfactory contact and cooling. Gross device readjustment can be performed at this stage if either the UA or ECD position or orientation is not satisfactory.

**Fig. 4:**
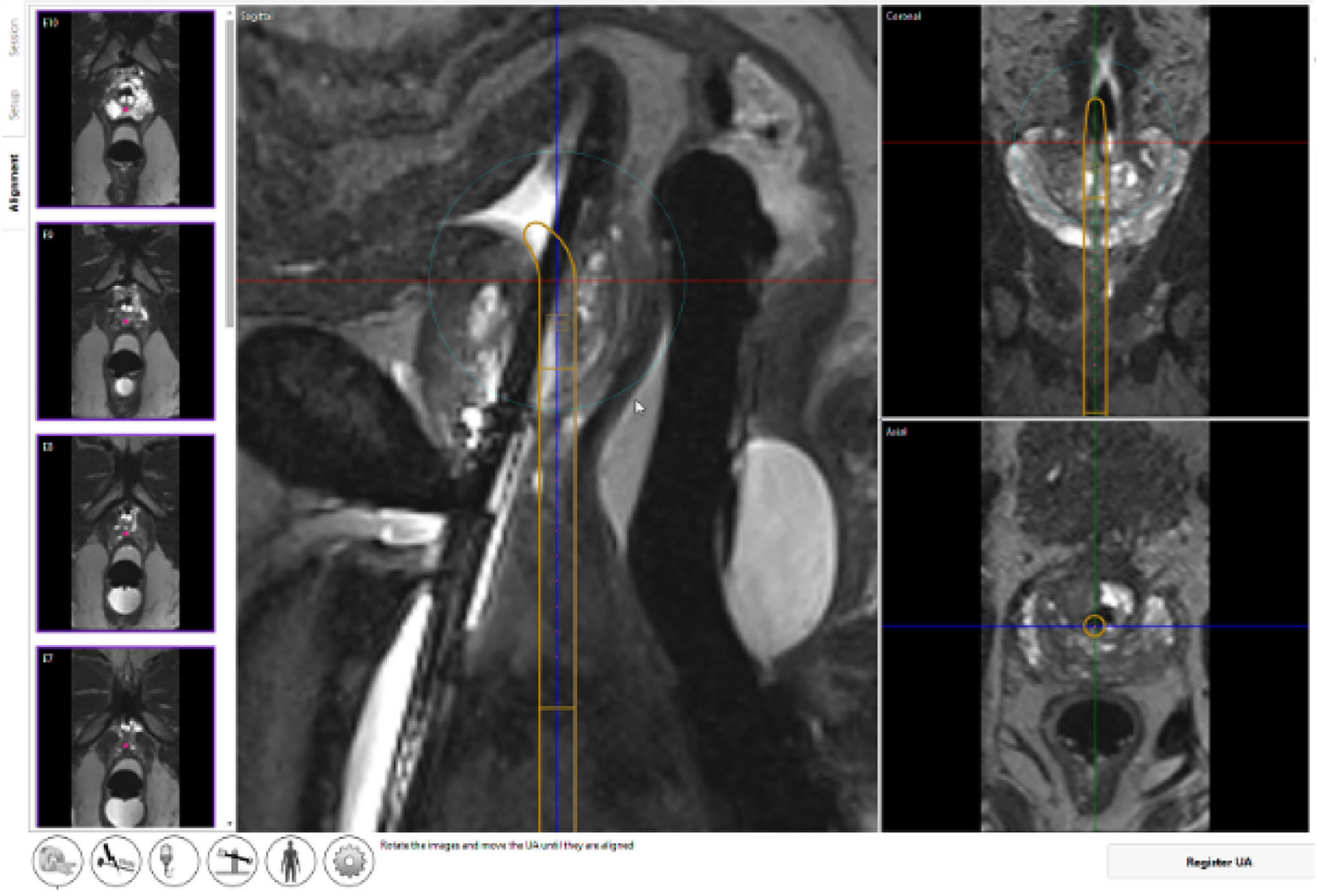
Device registration. The aim is to align the graphical representation of urethral applicator (orange line) with the actual physical position of urethral applicator on all three planes of MR images (represented by the cross hairs in red, blue and green lines). This allows subsequent ablation energy to be delivered accurately within the prostate

When both UA and ECD are appropriately placed, device registration and alignment can be performed (Figure 4, Supplementary video 1). Three-dimensional T2-weighed images would be acquired and imported to the treatment delivery console, where the radiologist can register the location and angulation of the UA on the console in all three planes. The robotic system can assist with fine adjustment of UA position along the linear axis at this stage.

- (b) Planning

The radiologist needs to verify that (i) the active US transducers are covering the target prostatic tissue to be ablated and (ii) a 4-mm safety margin was present between the active transducer and the external sphincter and bladder neck, respectively [13]. Depending on the craniocaudal dimension of the prostate gland, the radiologist can select the number of US transducers to be activated.

**Fig. 5:**
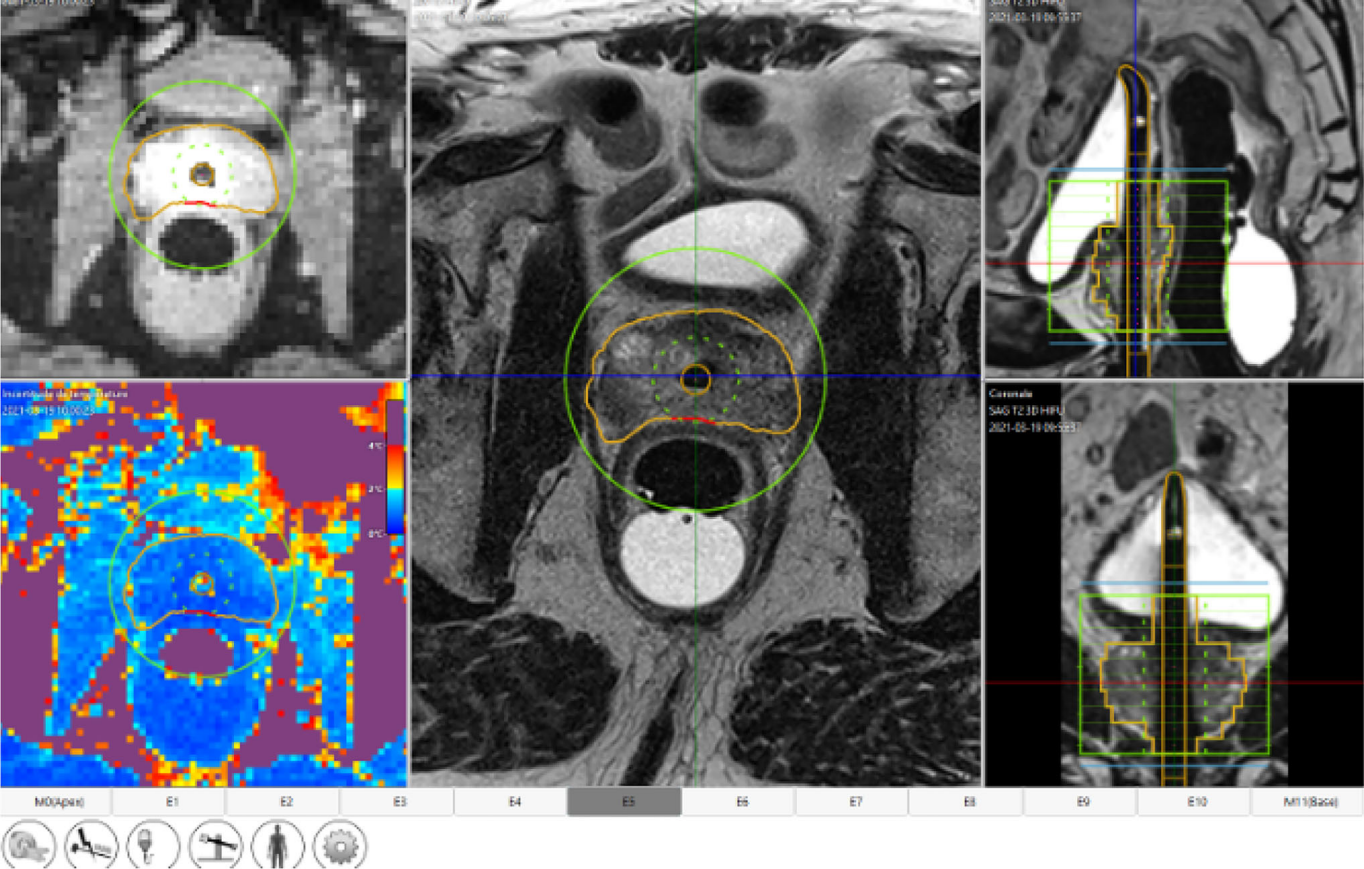
Treatment planning. The radiologist can draw the desired treatment boundary (orange line) on the axial T2-weighed MR image for each transducer element separately. The transducer within 3 mm of urinary bladder neck and external urinary sphincter can be deactivated to prevent thermal injury. The quality of MR thermometry can also be evaluated at this stage. The colour on the MR thermometry quality map (left lower corner) represents change in temperature from baseline, with dark blue indicating 0 °C change, i.e. good quality, and dark red indicating 4 °C change, i.e. suboptimal quality

Subsequently, high resolution T2-weighed axial and MR thermometry images which are perpendicular to the UA axis are obtained. Based on these two sequences, the radiologist can contour the targeted prostatic boundary for each element (Fig. 5). The software would alert the radiologist if the contour drawn is beyond the reachable limit of US beam or if the MR thermometry in the region is unreliable. Care should be taken to include the MR-visible lesions while avoiding the rectal wall, external sphincter, bladder neck and neurovascular bundles. The combined set of axial boundaries from each active transducer would define the 3-dimensional volume of the prostate gland to be ablated.

It is important to note that MR thermometry during TULSA utilises the proton resonance frequency (PRF) method for estimation of tissue temperature. The PRF method derives the tissue temperature by measuring phase difference in proton resonance frequency resulting from temperature-dependent change, i.e. it is a relative temperature measurement versus the baseline [19]. Therefore, any motions during real-time MR thermometry would result in misregistration artefact and lead to error in temperature measurement. General anaesthesia with muscle relaxation is hence mandatory during TULSA to prevent patient motion. In addition, PRF MR thermometry is only accurate in water-based tissue as the resonance frequency of proton in fat is not temperature dependent [19], hence MR monitoring of temperature in periprostatic fat is not possible during TULSA.

- (c) Delivery

**Fig. 6:**
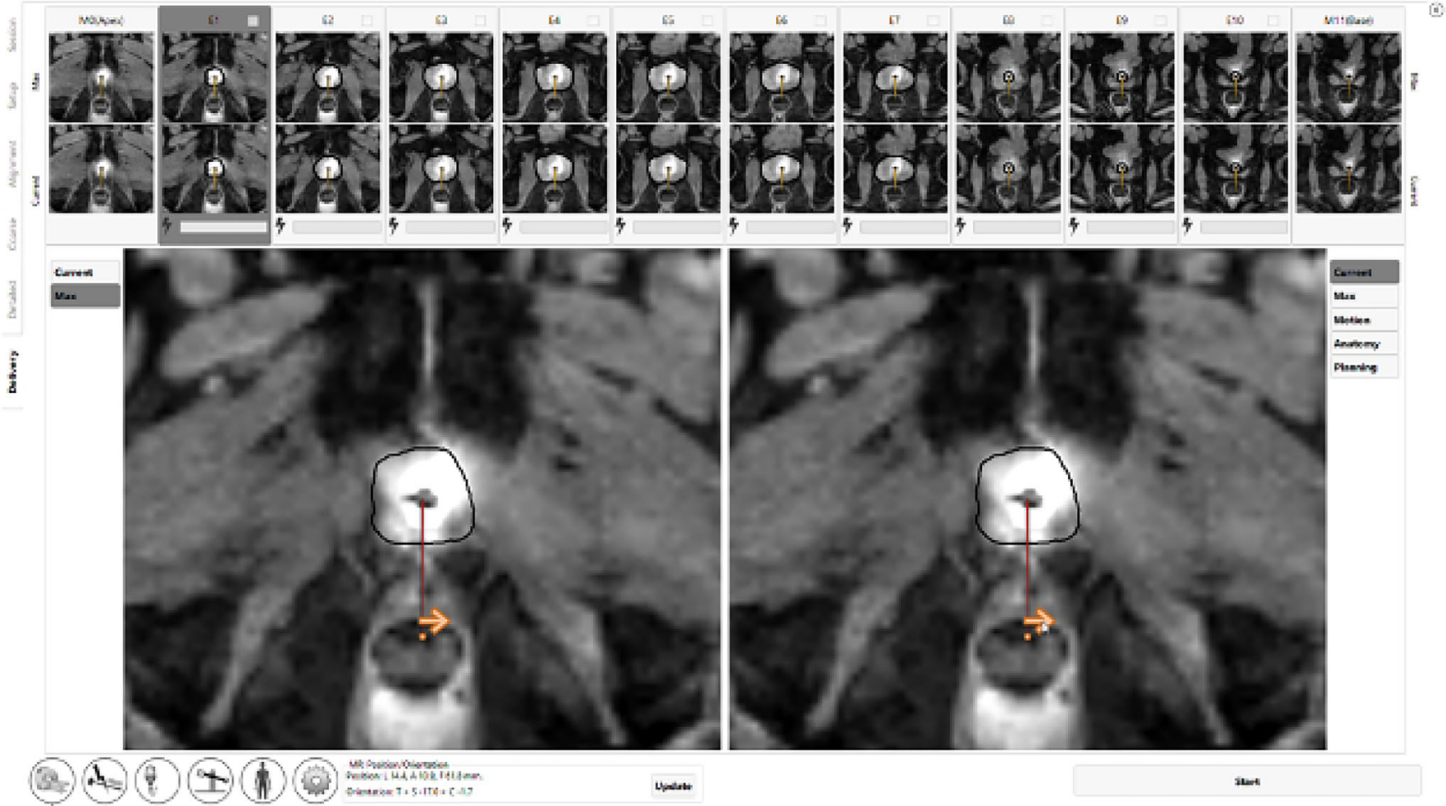
Initiation of treatment delivery. The radiologist can choose the point and direction to initiate the angular sweep of the US beam, as indicated by the arrow

The radiologist can decide where to initiate the angular sweep of directional US beam. Usually this is done a few degrees before the target lesion, with the beam subsequently sweeping in clockwise or anti-clockwise direction (Fig. 6, Supplementary video 2). It is important to note that, at the initial stage of ablation, the directional US beam from each transducer would be at its full power. Therefore, it would be prudent to avoid place the starting point of ablation sweep at the rectal region.

**Fig. 7:**
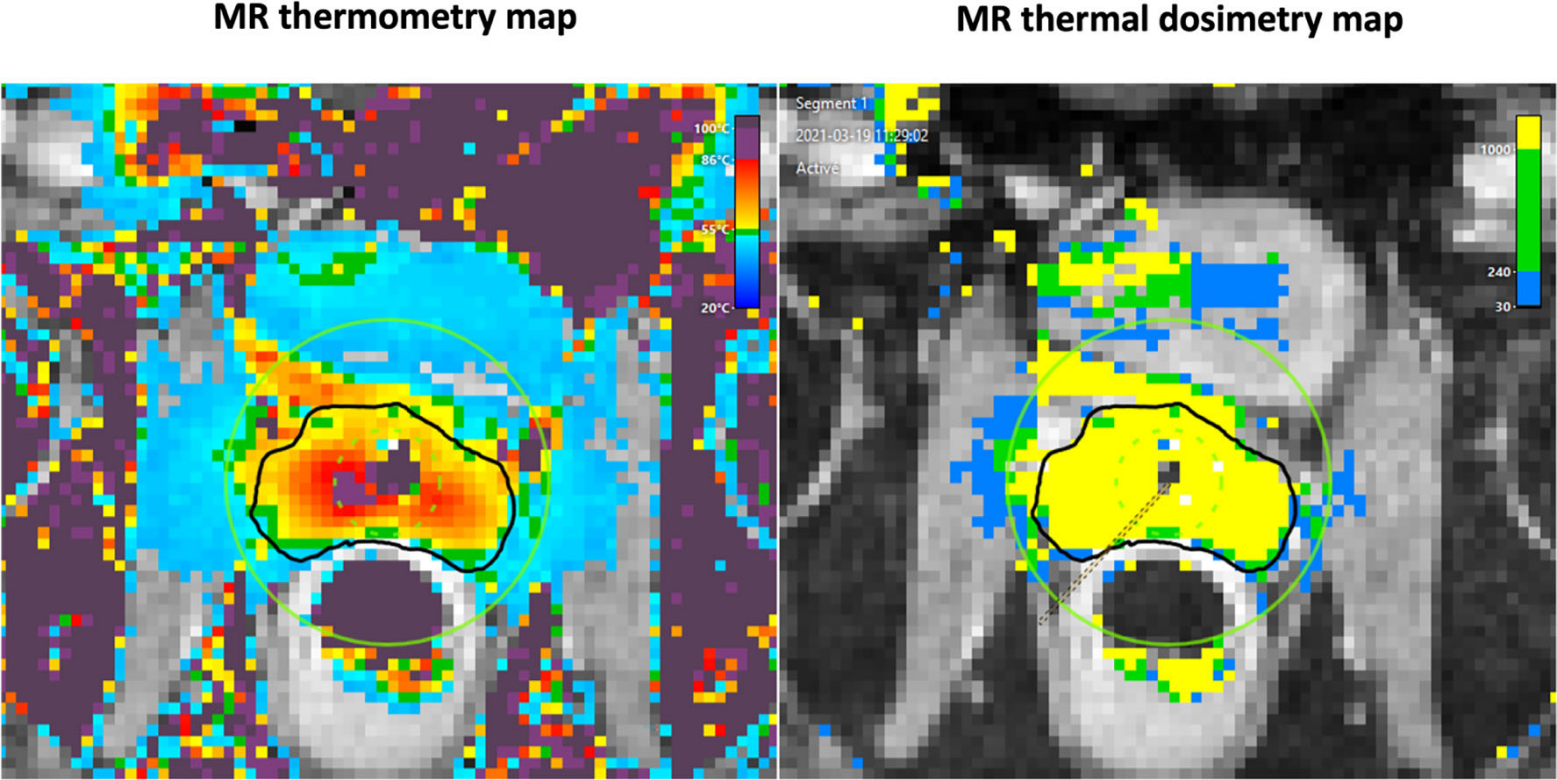
Real-time monitoring of treatment delivery. The radiologist can view real-time temperature and thermal dose (in units of CEM43, i.e. cumulative equivalent minutes at 43 °C) delivered in the prostate and surrounding areas. Please note the artefact within the urinary bladder (white arrow) which does not correspond to the true delivered dose. This artefact occurs as a result of fluid motion of urine

Once the ablation begins, the power of directional US beam would be modulated under the guidance of real-time MR thermometry and a closed-loop feedback temperature control system to avoid under-heating or over-heating. The radiologist can view the real-time temperature and thermal dose map on the treatment delivery console and adjust the treatment boundary if necessary (Fig. 7). The tissue heated to 55 °C, or a thermal dose exceeding 1000 CEM 43 (cumulative equivalent minutes at 43 °C), results in acute thermal coagulation. The temperature of 52 °C, or a thermal dose of 240 CEM 43, has approximately 1–3-mm margin of delayed cell kill [20–22]. The US beam power can also be boosted temporarily in case the radiologist deems the ablation margin needs to be extended beyond its original boundary or additional thermal dose is required to be delivered to an “index lesion”. Ablation treatment can also be paused in case anaesthetic personnel needs to enter the MR suite. However, at re-initiation of treatment, the US beam would be again at its full power.

- (d) Confirmation

**Fig. 8:**
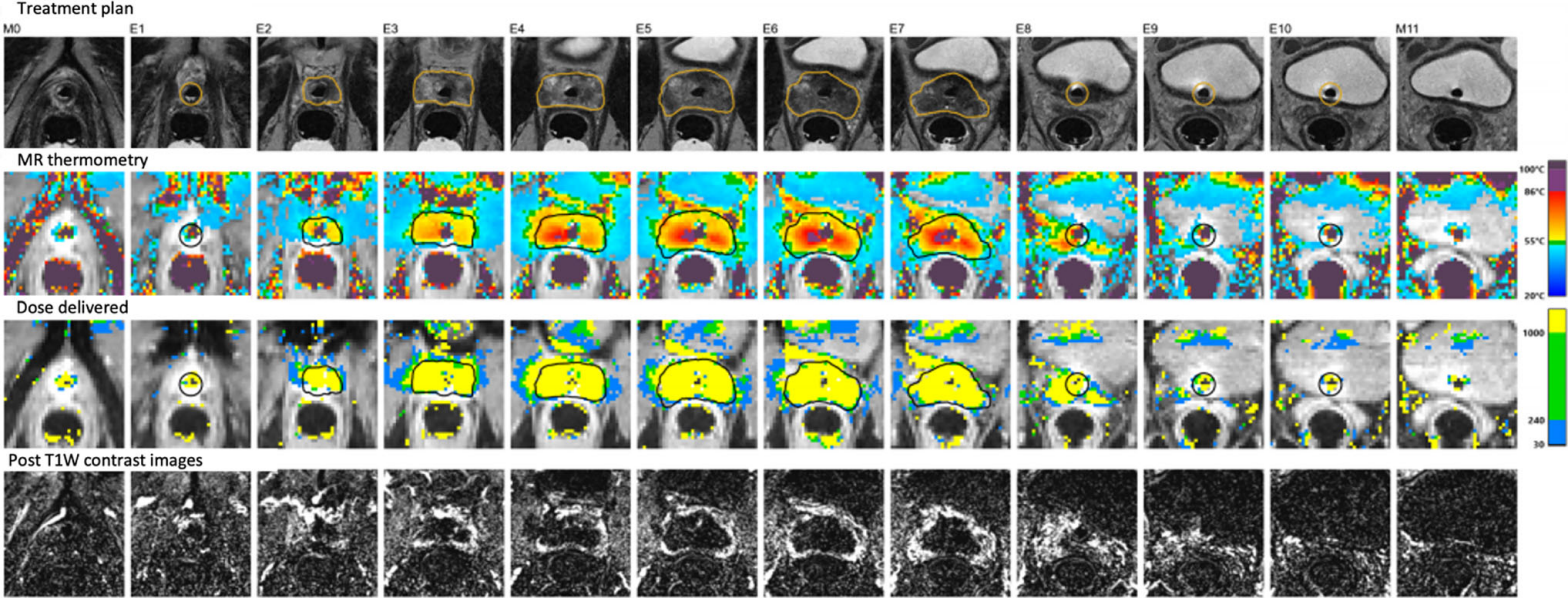
Treatment confirmation. The summary images show treatment delivered at each transducer level of the urethral applicator. The non-perfused prostatic volume shown on T1-weighed post-contrast subtraction images (bottom row) confirms extent of ablation

At treatment completion, post-gadolinium contrast enhanced T1-weighed images would be acquired to assess the non-perfused area in the prostate gland, which corresponds to the ablation extent (Fig. 8). Foley’s catheter would be reinserted and kept for 24 h before removal.

## Current evidence and way forward

The optimal strategy for low- and intermediate-risk localised prostatic cancer, especially for men who prefer curative treatment over active surveillance, is unclear [2–5]. Ablative modalities including cryoablation, focal photodynamic therapy, transrectal/MR-guided HIFU and TULSA have been developed to improve functional preservation without compromising oncological outcome [23–25]. Compared to other ablative modalities, TULSA has the following benefits: (i) ability to deliver focal/partial and whole-gland ablation, (ii) real-time modulation of ablation intensity under MR thermometric guidance, (iii) an “inside-out” approach, i.e. the thermal energy is delivered from urethra to the prostatic capsule, avoiding direct thermal contact with periprostatic neurovascular bundles.

Table 1 summarises the prospective trials for MR-guided whole-gland TULSA in the literature for treatment of primary localised PCa. The largest pivotal prospective single-arm multi-centre trial (TACT) assessed the short- and mid-term outcome of whole-gland TULSA in 115 patients with low- to intermediate-risk PCa [13] (Klotz et al. Abstract Mp46-03. Journal of Urology. 2021 Sep; 206 S3: e814–e814.) In terms of treatment efficacy after single TULSA procedure, 96% (110/115) of patients achieved PSA reduction of 75% or greater. Median PSA reduction was 95% to a median nadir of 0.34 ng/ml at 1 year and 0.26 ng/mL at 3 years. Reassessment MRI and prostate biopsy performed at 1 year showed that 65% (72/111) of patients had no evidence of cancer. At 3-year follow-up of the pivotal study, 13% (15/115) underwent salvage treatment, including radical prostectomy and external beam radiotherapy.

**Table 1:** Summary of prospective trials evaluating the treatment efficacy and safety of whole-gland TULSA in primary localised PCa [13, 15, 16]

Study	Design	Indications	Follow-up (months)	Inclusion criteria	Number of patients	Median age (years)	Oncological outcome				Functional outcome			Complications
							Baseline median PSA, ng/mL (IQR)	% Reduction of median PSA	Median PSA at follow-up, ng/mL (IQR)	Number of patient requiring salvage treatment (%)	% Patient with preserved baseline potency, i.e. IIEF Q2 ≥ 2	% Patient with preserved baseline continence	% Reduction in IPSS score	
Chin et al. [15]	Single-arm, prospective, phase 1, safety and feasibility multi-centre (not intention-to-treat)	Whole-gland ablation in men with low- to intermediate-risk localised PCa	12	Treatment-naive men ≥ 65 year Biopsy-proven T1c–T2a, N0, M0 prostate cancer PSA ≤ 10 ng/ml, Gleason score 3 + 3 or 3 + 4	30	69	5.8 (3.8–8.0)	86.2	0.8 (0.6–1.1)	2	85% (17/20)	100% (30/30)	38% (Baseline = 8, follow-up = 5)	Haematuria = 13 (43%) Urinary tract infections = 10 (33%) Acute urinary retention = 3 (10%) Epididymitis = 1 (3.33%)
Nair et al. [16]			36		22			86.2	0.8 (0.6–1.6)	7	85% (11/13)	100% (22/22)	0% (Baseline = 6, follow-up = 6)	
Nair et al. [30] (Abstract only)			60		16			90.5	0.6 (0.4–12)	10	77.8% (7/9)	100% (16/16)	25% (Baseline = 8, follow-up = 6)	
Klotz et al. [13]	Prospective, multi-centre, single-arm pivotal (intention-to-treat)	Whole-gland ablation in men with low- to intermediate-risk localised PCa	12	Age 45 to 80 years Gleason Grade Group 1–2 Clinical stage ≤ T2b PSA ≤ 15 ng/ml Minimum 10-core biopsy No previous treatment	115	65	6.3 (4.6–7.9)	92.0	0.5 (0.3–12)	8	75% (69/92)	92% (103/112)	14% (Baseline = 7, follow-up = 6)	Genitourinary infections = 5 (4%) Urethral stricture = 2 (2.3%) Urinary retention = 2 (1.7%) Urethral calculus = 1 (1%) Pain = 1 (1%) Urinoma = 1 (1%)
Klotz [13] (Abstract only)			36					89.0	0.7 (N/A)	15	80% (40/50)	97.4%	43% (Baseline = 7, follow-up = 4)	

In terms of functional outcome, initial deterioration of urinary, bowel and sexual function was observed at 1 month but most return to baseline by 3–6 months. For men who were potent at baseline with 3-year follow-up, 72% (36/50) maintained or regained erection firmness sufficient for penetration at 1 year and 80% (40/50) at 3 years. 2.6% (3/111) patients experienced moderate urinary incontinence at one year, with one patient persisting into three years. Comparing to the functional outcome of conventional treatments of favourable-risk PCa in a prospective population-based cohort study [26], only 48% patients maintained or regained erection firmness at 5 years after nerve-sparing radical prostatectomy and 54% after low-dose rate brachytherapy.

In terms of safety, a total of 12 Grade 3 (severe) adverse events occurred in 8% (9/111) men, including genitourinary infection (4%), urethral stricture (2%), urinary retention (1.7%), urethral calculus and pain (1%) and urinoma (1%), all of which were resolved by 1 year. There were no Grade 4 events, rectal injuries, severe incontinence requiring surgical intervention or severe erectile dysfunction unresponsive to medication.

A recent systematic review on MR-guided TULSA identified four studies (including the pivotal trial described above, including both focal and whole-gland TULSA) which investigated the aggregate outcome of a total of 198 patients with primary localised PCa [27]. After a single TULSA treatment, the rate of salvage treatment ranged from 7 to 17% by median of 16 to 24 months. It is, however, important to note that most of TULSA studies included patients with low-risk localised PCa, who have lower rates of metastasis and prostate cancer related death [26]. This may lead to bias of results in favour of TULSA.

Based on the promising results from large scale prospective trials, whole-gland TULSA has an increasing role in treatment of primary localised PCa. A few smaller scale studies have also explored its efficacy and safety in other indications, including focal/partial gland ablation in low-risk primary PCa, palliation for men with symptomatic locally advanced PCa, salvage treatment for localised radio-recurrent PCa and benign prostate hypertrophy. Table 2 summarises the evidence in the literature for TULSA in these indications [17, 28–31]. While the initial results are encouraging, these studies are mainly small-scale feasibility trials or retrospective in design. There is also a lack of long term follow-up data in these studies.

**Table 2:** Summary of current evidence of MR-guided TULSA in indications other than whole-gland ablation for primary localised PCa [17, 28–31]

Study	Design	Indications	Follow-up (months)	Inclusion criteria	No. of patients	Median age (years)	Oncological outcome				Functional outcome	Complications
							Baseline median PSA, ng/mL (IQR)	% reduction of median PSA	Median PSA at follow-up, ng/mL (IQR)	Recurrence		
Anttinnen et al. (2020)	Prospective, single centre, phase I study	Salvage TULSA in radio-recurrent PCa	12	Pathologically proven localised PCa which failed radiotherapy	11	69	7.6 (4.9–10)	97%	0.23 (0.2–0.9)	2 out-of-field recurrence 1 in-field recurrence (Biopsy proven)	All men had severe erectile dysfunction at baseline Minimal decrease in EPIC score for urinary continence (Baseline = 100; 12 months follow-up = 96)	Urinary retention = 3 (25%) Urinary tract infection = 4 (33%)
Anttinnen et al. (2020)	Single centre, prospective, phase I study	Palliative control for locally advanced PCa	12	Primary or radio-recurrent localised PCa (with or without metastasis) Haematuria and/or urinary retention requiring continuous catheterisation, which were unresponsive to conservative treatment Expected life expectancy ≥ 3 months	10	76.5	N/A	N/A	N/A	N/A	50% patients are catheter-free at 1-week At 12 month follow-up, 70% patients are catheter-free and 100% patient are gross hematuria-free	Urinary tract infection = 3 (33%)
Elterman et al. [30]	Retrospective subgroup analysis of multi-centre Phase I trial	Control of LUTS in men with concurrent BPH and PCa	12	Same criteria as Chin et al. in Table 1 IPSS score ≥ 12 Qmax (Peak urinary flow) ≤ 15 ml/s	9	70.9	N/A	N/A	N/A	N/A	58% reduction in IPSS (Baseline = 16.1 ± 3.8; 12 months follow-up = 6.3 ± 5.0) 60% increase in Qmax (Baseline = 14.5 ± 4.1 mL/s; 12 months follow-up = 21.9 ± 12.7 mL/s)	Urinary tract infection = 3 (33%) Urinary retention = 3 (33%)
Lumiani et al. [31]	Retrospective, single centre, clinical service report	Focal/partial ablation for primary localised PCa	Median = 16, IQR = 12–22	Biopsy-proven low- and intermediate-risk localised PCa For focal TULSA, MRI visible lesion (PIRADS ≥ 3)	38	68	8.3 (5.0–12)	78	1.8 (1.0–3.1)	29% (11/38) had biochemical recurrence	100% patients maintained baseline full potency 98% patients had pad-free continence 83% patients in subgroup seeking BPH treatment experience symptomatic improvement	Acute urinary retention = 6 (12%) Urinary tract infection = 2 (3.8%) Prostatitis = 2 (3.8%) Epididymitis = 1 (1.9%) Haematuria = 1 (1.9%) Scrotal swelling = 1 (1.9%)
		PCa with BPH subgroup			24		N/A	N/A	N/A	N/A		
		Salvage TULSA in radio-recurrent PCa			5	71	7.0 (6.1–8.0)	54	3.2 (0.5–4.0)	40% (2/5) requiring had biochemical recurrence		
Peters et al. [17]	Retrospective, single centre	Focal/partial ablation for primary localised PCa	12	Interdisciplinary selection by urologist & radiologist Biopsy-proven low- and intermediate-risk localised PCa (ISUP grade 1 or 2) Prostate radius ≤ 3 cm	16	67	7.1 (5.4–8.1)	59.2	2.9 (1.5–3.7)	4 out-of-field recurrence 2 in-field recurrence (Biopsy proven)	No significant change in EPIC score for urinary continence (Baseline = 89 ± 19%; 12 months follow-up = 90 ± 5%) Erectile function significantly decreased compared to baseline (Baseline = 69 ± 6%)	Acute urinary retention = 1 (6%) Pelvic pain = 1 (6%)
		Salvage whole-gland TULSA in radio-recurrent PCa			2		7.0 (5.8–8.3)	99	0.07 (0.065–0.075)	Nil		

The main reasons for TULSA failure are insufficient thermal coverage around target lesion, calcifications disrupting the beam path and tumour locating beyond the reach of US beam. Large prostatic calcification (> 1 cm) can cause distortion of local MR thermometry and heating pattern, i.e. the calcification would be heated locally to up to 100 °C while the shadowed tissue would not be adequately heated. Presence of intraprostatic calcification was shown to be an independent predictive factor of persistent Gleason Grade 2 disease at 1 year follow-up in the pivotal trial [13]. This highlights the need of imaging protocol to identify intraprostatic calcification before TULSA, using susceptibility weighed sequences in MR or pre-TULSA CT scan [14] (Lee et al. Abstract No. 160. Journal of Vascular and Interventional Radiology. 2023 Mar 1; 34[3]: S74–5).

While the results from initial trials for whole-gland TULSA in treatment of primary localised PCa are promising, the current literature lacks high-quality evidence showing a direct head-to-head comparison between whole-gland TULSA and conventional treatment. The ongoing CAPTAIN trial (NCT05027477) should soon provide level I evidence concerning treatment efficacy and safety outcome of whole-gland TULSA vs radical prostatectomy for patients with localised PCa. In addition, the trial should provide us with more insight on who are the best patients to receive MR-guided whole-gland TULSA. In addition, for patients who failed initial MR-guided whole-gland TULSA, further research needs to be conducted to identify the optimal second-line salvage therapy. Of note, radical prostatectomy is a viable and safe salvage option if TULSA fails [32].

Limitation of MR-guided TULSA includes the initial capital investment in the MR-guided robotic system, need for general anaesthesia, MR-compatible anaesthetic equipments, relatively long procedural time (around 4 h from positioning of anaesthetised patient to recovery) and its inability to treat certain lesions, including those located within 3 mm of the urethra or urinary sphincter, in a prostate with calcification or cyst, or in a prostate larger than 6.0 cm in sagittal dimension or 5.0 cm in axial dimension [13]. These limitations are not unique to TULSA and exist in other ablative modalities as well.

Other novel MR-guided technologies in treatment of localised PCa include MR-guided HIFU (MRgFUS) and MR-guided radiation therapy (MRIgRT). MRgFUS utilises a transrectal applicator to deliver focused US beam to the prostate gland. Similar to MR-guided TULSA, HIFU ablation of the target area is monitored under real-time MR thermometry with a closed-loop feedback temperature control system. Two recent prospective multi-centre trials have demonstrated satisfactory oncological control with similar functional outcome compared to MR-guided TULSA [33, 34]. In MRIgRT, due to improved visualisation of soft tissue structures, radiation beam can be accurately delivered to the target prostatic volume while sparing critical structures including urethra, urinary sphincter, periprostatic neurovascular bundles and penile structure [35]. The ongoing ERECT trial (NCT04861194) aims to evaluate mid-term erectile function in neurovascular-sparing MRIgRT.

In conclusion, MR-guided whole-gland TULSA is an emerging ablative modality in the management of localised PCa at its initial or recurrent setting, with good oncological outcome and reduced impairment of genitourinary function compared to radical prostatectomy or external beam radiotherapy. Ongoing randomised controlled trial will provide high-quality evidence to further establish the role of whole-gland TULSA in treatment of localised PCa. Additional high-level evidence is needed in evaluating the use of focal TULSA in localised PCa.

## Supplementary Information

Below is the link to the electronic supplementary material.

Supplementary file1 (MP4 11901 KB)

Supplementary file2 (MP4 57277 KB)
